# Effect of photoinitiator on chain degradation of hyaluronic acid

**DOI:** 10.1186/s40824-019-0170-1

**Published:** 2019-11-21

**Authors:** Bo Min Hong, Su A Park, Won Ho Park

**Affiliations:** 1 Department of Advanced Organic Materials and Textile System Engineering, College of Engineering, Chungnam National University, Daejeon, 34134 South Korea; 2 Department of Nature-Inspired Nanoconvergence Systems, Korea Institute of Machinery and Materials, Daejeon, 34103 South Korea; 3 Department of Advanced Organic Materials and Textile System Engineering, College of Engineerings, Chungnam National University, Daejeon, 305-764 South Korea

**Keywords:** Riboflavin, Hyaluronic acid, Tyramine, Hydrogel, Photocrosslinking, Degradation

## Abstract

**Objectives:**

Photocrosslinking systems of polymers have been widely studied using UV or visible light irradiation. However, the photodegradation behavior derived from light irradiation was rarely reported, comparing with the photocrosslinking. In this study, the tyramine-modified hyaluronic acid (HA/Tyr) hydrogel was prepared using riboflavin (RF) as a photoinitiator, and the degradation behavior of HA by the reactive oxygen species (ROS) generated in photochemical process was investigated.

**Materials and methods:**

The HA/Tyr conjugate was synthesized by EDC/NHS chemistry to introduce phenol group. Degree of substitution (DS, %) of phenol group to HA molecule was about 25%. The structural change of HA/Tyr was measured by proton nuclear magnetic resonance (^1^H-NMR) and attenuated total reflectance infrared spectroscopy (ATR-FTIR), and the rheological properties of photocrosslinked HA/Tyr hydrogel were investigated by rheometer.

**Results:**

The HA/Tyr solution with 25% substitution formed a stable hydrogel via visible light irradiation in the presence of RF photoinitiator. Rheological data of HA/Tyr solution showed that the storage modulus (G’) was increased with increasing HA concentration. Additionally, it was found that RF initiated by visible light irradiation induced the degradation of HA molecular chain, and consequently reduced the viscosity of HA/Tyr solutions.

**Conclusion:**

The results indicate that RF-based photoinitiator system caused the degradation of HA molecule by ROS generated in photochemical process as well as the crosslinking of HA/Tyr.

## Introduction

Hyaluronic acid (HA), or hyaluronan, is a linear natural polysaccharide composed of D-glucuronic acid and N-acetyl glucosamine. HA is a non-sulfated glycosaminoglycan (GAG), an essential component of the extracellular matrix (ECM) in many parts of the body, which exhibits excellent viscoelastic property, favorable biocompatibility, and biodegradability [1]. Additionally, HA is interactive and binds to many types of cell surface receptors containing CD44, ICAM-1, and RHAMM [2, 3]. Because of these unique properties of HA, it has been widely used in biomedical applications such as osteoarthritis, wound healing, drug delivery, and tissue engineering scaffolds [1, 4]. However, native HA has some limitations that it cannot last long in the human body due to its poor mechanical properties and rapid degradation by hyaluronidase in vivo. It has been reported that the half-life of HA does not exceed 1 day after injection into joints or skin [5]. To overcome these problems, HA should be crosslinked via various physical and chemical crosslinking methods to ensure a longer residence time with mechanical strength in the body. Particularly, the introduction of chemical crosslinking can generate a 3-D hydrogel network containing a large amount of water, and the resultant stable hydrogel retains a higher resistance to enzymatic degradation and molar mass reduction compared to native HA. In general, HA can be chemically crosslinked using crosslinking reagents such as 1,4-butanediol diglycidyl ether (BDDE) [6], divinyl sulfone (DVS) [7] and carbodiimide. In addition to direct chemical crosslinking of native HA containing carboxylic acid and hydroxyl groups, HA hydrogels can be also prepared by physical and chemical crosslinking of HA derivatives modified with various photofunctional groups. Recently, the chemically crosslinked HA hydrogel was fabricated via photocrosslinking using photoinitiators and visible/UV light [8–11]. Several studies reported that visible light can induce the gelation of tyramine (Tyr)-modified polymers in the presence of ruthenium (Ru (II)) photoinitiator and sodium persulfate (SPS) [12, 13]. Also, riboflavin (RF) and SPS as photoinitiators induce the gelation of polymers by visible light irradiation.

**Fig. 1:**
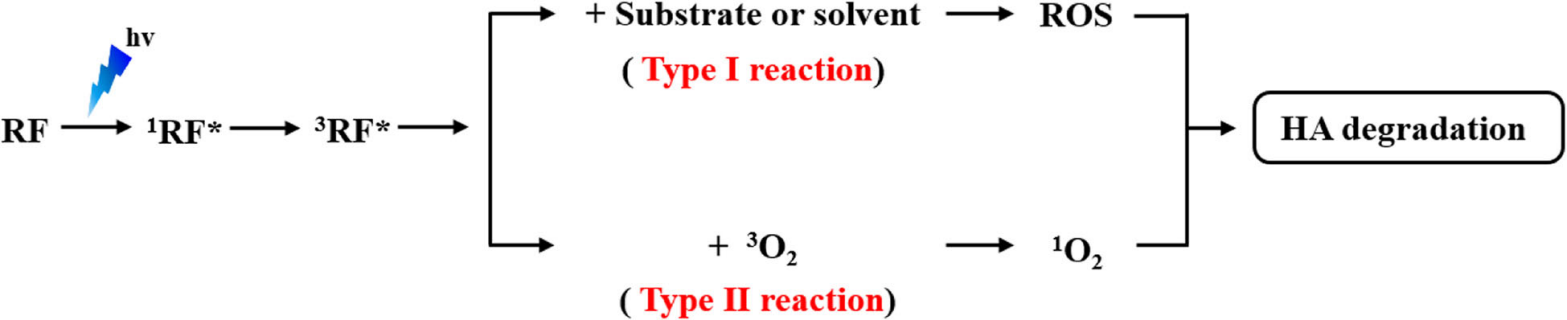
Excitation of RF and generation of ROS according to two possible photochemical reaction (Type I and II)

RF, known as vitamin B_2_, is a naturally occurring photoinitiator used in the manufacture of various photocrosslinked hydrogels. RF can be reversibly reduced and oxidized by accepting or losing a pair of hydrogen atoms (Redox reaction). The redox reaction of RF can induce the crosslinking reaction via radical formation [14]. The crosslinking mechanism involves the excitation of RF by light absorption at 220–450 nm wavelengths, that is, UV and visible region. In the photochemical reaction, RF absorbs light energy from UV or visible light and excited to short lived singlet state (^1^RF*, 10^− 8^ s lifetime), which transformed into a highly reactive and long lived triplet-excited state (^3^RF*, 10^− 2^ s lifetime). ^3^RF* is a powerful oxidant biradical. In the presence of oxygen, ^3^RF* can transfer energy directly to molecular oxygen in the ground state (^3^O_2_) to produce reactive singlet oxygen (^1^O_2_). These reactions are commonly referred to as type II reaction (energy transfer). Alternatively, ^3^RF* can react with other substrates or solvents, and can generate free radicals or radical ions by hydrogen atom extraction or electron transfer to the substrate. These radicals can interact with ^3^O_2_ to produce the reactive oxygen species (ROS) such as superoxide anion radicals (O_2_^∙-^), hydrogen peroxides (H_2_O_2_), hydroxyl radicals (∙OH), etc., and these reactions are commonly referred to as type I reaction (electron-transfer) (Fig. 1) [15–18]. Both these reactions occur simultaneously.

In this study, the photocrosslinked HA/Tyr hydrogel was prepared using RF-based initiators under visible light irradiation. Visible light was used as an alternative irradiation source of UV light because it has longer wavelengths, and thus makes more preferable condition for living cells and desirable for biomedical applications [19–21]. In this photo-crosslinking process, together with the oxidation, the dityrosine crosslinking was formed by the reaction between tyrosil radicals generated from phenolic groups (tyramine moiety) of HA/Tyr. Notably, RF-initiated HA/Tyr can also cause the chain degradation of HA by ROS (including ^1^O_2_) as well as crosslinking via the formation of dityrosine. Therefore, the effect of photosensitized RF and generated ROS on the HA molecular chain was investigated using viscosity change of HA/Tyr solution.

## Materials and methods

### Materials

Hyaluronic acid (HA, sodium salt form, Mw 550 kDa) was purchased from Shandong Freda Biochem Co. Ltd. (Jinan, China). 1-Ethyl-3-(3-dimethylaminopropyl) carbodiimide hydrochloride (EDC), N-Hydroxysulfosuccinimide (NHS) and sodium persulfate (SPS) were supplied by Sigma-Aldrich Co. (St Louis, MO, USA). Riboflavin 5′-phosphate sodium salt (RF) was supplied by Tokyo Chemical Industry Co. Ltd. (Tokyo, Japan). Tyramine hydrochloride (Tyr) was supplied by Acros Organics (Morris Plains, NJ, USA).

### Synthesis of HA/Tyr conjugate

Tyramine-modified hyaluronic acid (HA/Tyr) was synthesized by coupling HA with tyramine hydrochloride via EDC/NHS coupling reaction. Briefly, HA sodium salt (1 g, 2.5 mmol) was dissolved in 100 ml of distilled water to obtain 1 wt% HA solution. To activate the carboxyl group of HA, EDC (0.48 g, 2.5 mmol) and NHS (0.29 g, 2.5 mmol) were added to the HA solution and reacted for 5 min in an ice bath. After this time, Tyr (0.717 g, 1.25 mmol) was dissolved in HA solution and reacted for 1 h in the ice bath. The solution was stirred overnight at room temperature. The reaction mixture was purified by dialysis using cellulose membrane (cut-off from 120,000 to 13,000) for 3 days. The resulting HA/Tyr conjugate was collected as solid powder after lyophilization [22–24].

Conjugation of tyramine to the carboxylic acid of HA was confirmed by ^1^H-NMR (600 MHz, ADVANCE III, Bruker, USA). The degree of substitution (DS, %) of Tyr residues in HA/Tyr conjugate was defined as a number of tyramine molecules per 100 repeating units of HA, and calculated by comparing the ratio of the relative peak integration of the tyramine aromatic protons (δ 6.8–7.2 ppm) and acetyl methyl protons of HA (δ 2.0 ppm) using the following equation. The degree of substitution was approximately 25%.

1 $$ \mathrm{DS}\ \left(\%\right)=\frac{I_{Tyr}/4}{I_{HA}/3}\times 100 $$

### Preparation of HA/Tyr hydrogels using RF and visible light

HA/Tyr hydrogel was prepared via photocrosslinking of the phenolic moieties induced by RF and visible light irradiation [25]. The concentration range of HA/Tyr solutions was 0.5–2.0 wt%. 0.5 mM of RF and 100 mM of SPS were added into the HA/Tyr solution, and stirred for 5 min [8]. The visible light (2500 mW/cm^2^, 440 nm) was irradiated for 30 s, and the hydrogel was kept overnight to complete the crosslinking reaction.

### Structural and rheological analyses

ATR-IR spectroscopy (ALPHA-P, Bruker, USA) of HA/Tyr sample was conducted to confirm the introduction of Tyr to the HA backbone. Rheological measurement was performed using a rheometer (MARS 40, Haake, Germany) with a parallel plate geometry (60 mm diameter and 1 mm gap). The parameters were determined, based on the amplitude sweep in the linear region of storage modulus (G’) and loss modulus (G”) to find range in which polymer can maintain structural stability without deformation (shear stress, τ = 1 Pa). Then, frequency sweep of hydrogel was measured to examine the gel strength with a 1 Pa and 37 °C from 0.1 to 10 Hz. Also, a time sweep of hydrogel was performed to confirm a stable gel formation during 900 s at 37 °C. To investigate the effect of ROS on the HA chain degradation, the viscosity change of HA solution in the presence of RF and SPS was measured in dark and light conditions.

## Results

### Synthesis and characterization of HA/Tyr conjugate

**Fig. 2:**
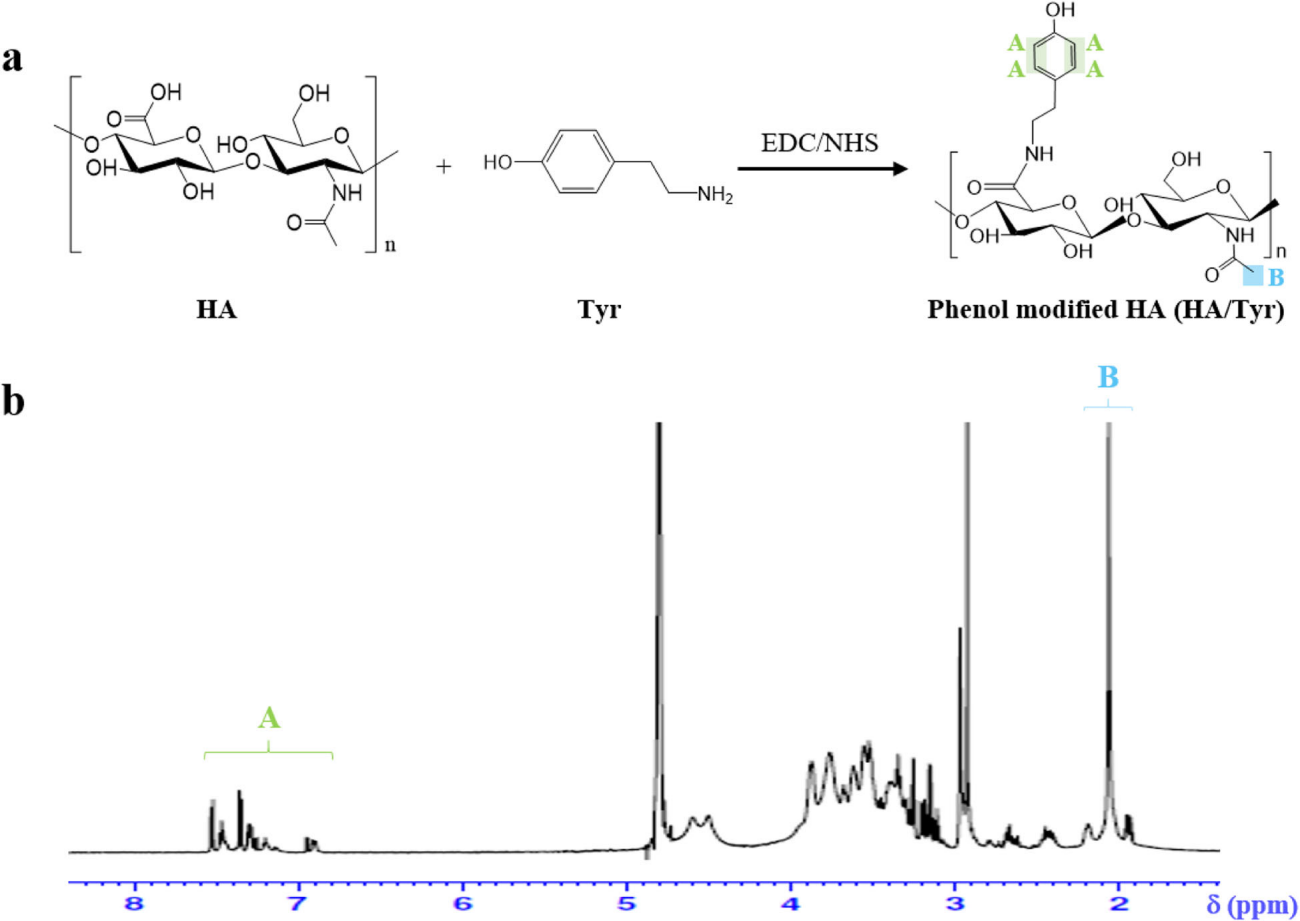
Chemical modification of Tyr on HA backbone. **a** Synthesis of HA/Tyr conjugates via EDC/NHS coupling reaction, **b** ^1^H-NMR spectrum of HA/Tyr 25

HA/Tyr conjugate was synthesized by introducing tyramine moiety to the HA backbone by carbodiimide coupling reaction using EDC/NHS. The coupling reaction occurred between carboxylic group of HA and amino group of tyramine (Fig. 2a). The DS (numbers of tyramine molecules per 100 repeating units of HA molecule) was determined by comparing the relative peak ratios of the 3 acetyl methyl protons of HA (2.0 ppm) and the 4 phenyl protons of tyramine (6.8–7.2 ppm) (Fig. 2b), using ^1^H-NMR spectrum. In this reaction, the molar ratio of Tyr to HA backbone was 0.5:1, the DS of HA/Tyr was ~ 25%, and it was named as HA/Tyr 25. The ^1^H NMR suggested that the HA/Tyr conjugate was successfully synthesized.

### Formation of photocrosslinked HA/Tyr hydrogels

**Fig. 3:**
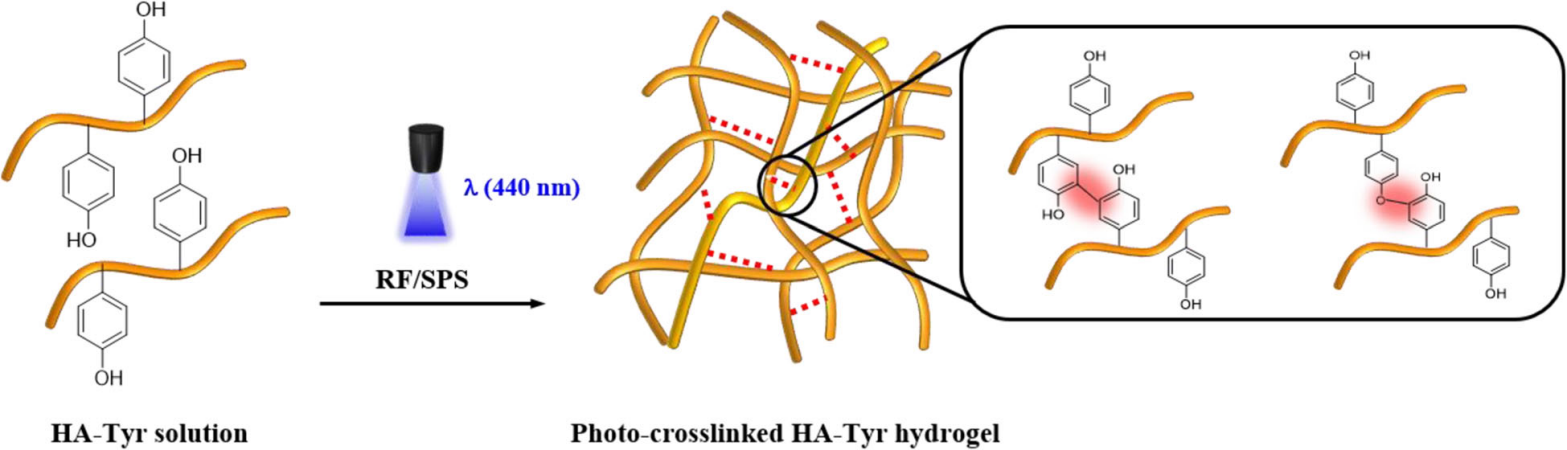
Schematic presentaion of HA/Tyr 25 hydrogel induced by visible light-irradiated crosslinking reaction using RF/SPS

**Fig. 4:**
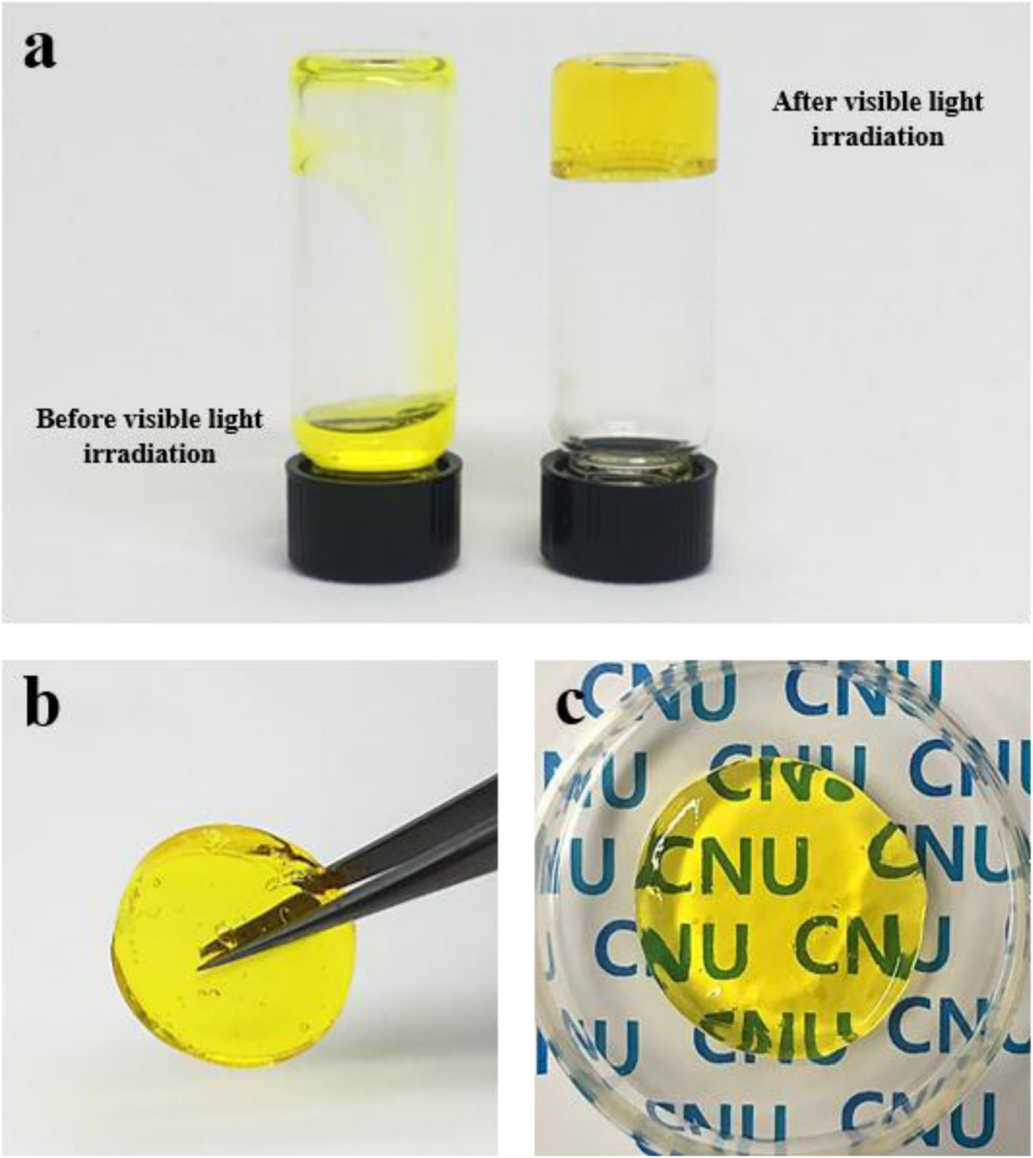
Photographs of photocrosslinked HA/Tyr hydrogel, **a** HA/Tyr solution before (left) and after (right) visible light irradiation for 30 s, **b** photograph showing easy handling of the HA/Tyr hydrogel with surgical forceps, and (**c**) transparency of the HA/Tyr hydrogel

Figure 3 shows a visible light-induced crosslinking mechanism of the HA/Tyr conjugate using RF photoinitiator. When visible light (λ = 440 nm) was exposed in the presence of RF, RF excited to ^3^RF*, and ^3^RF* induced the crosslinking reaction between phenolic groups of conugated tyramines. Then, the HA/Tyr solution immediately started to form gel. Photo-sensitive sol-gel transition behavior of HA/Tyr was observed by a tube inverting method (Fig. 4a). The aqueous solution of HA/Tyr was transformed to a yellowish transparent hydrogel with no fluidity, and the gel was able to be picked up with surgical forceps (Fig. 4b,c).

### Gelation behavior of photo crosslinked HA/Tyr conjugate

**Fig. 5:**
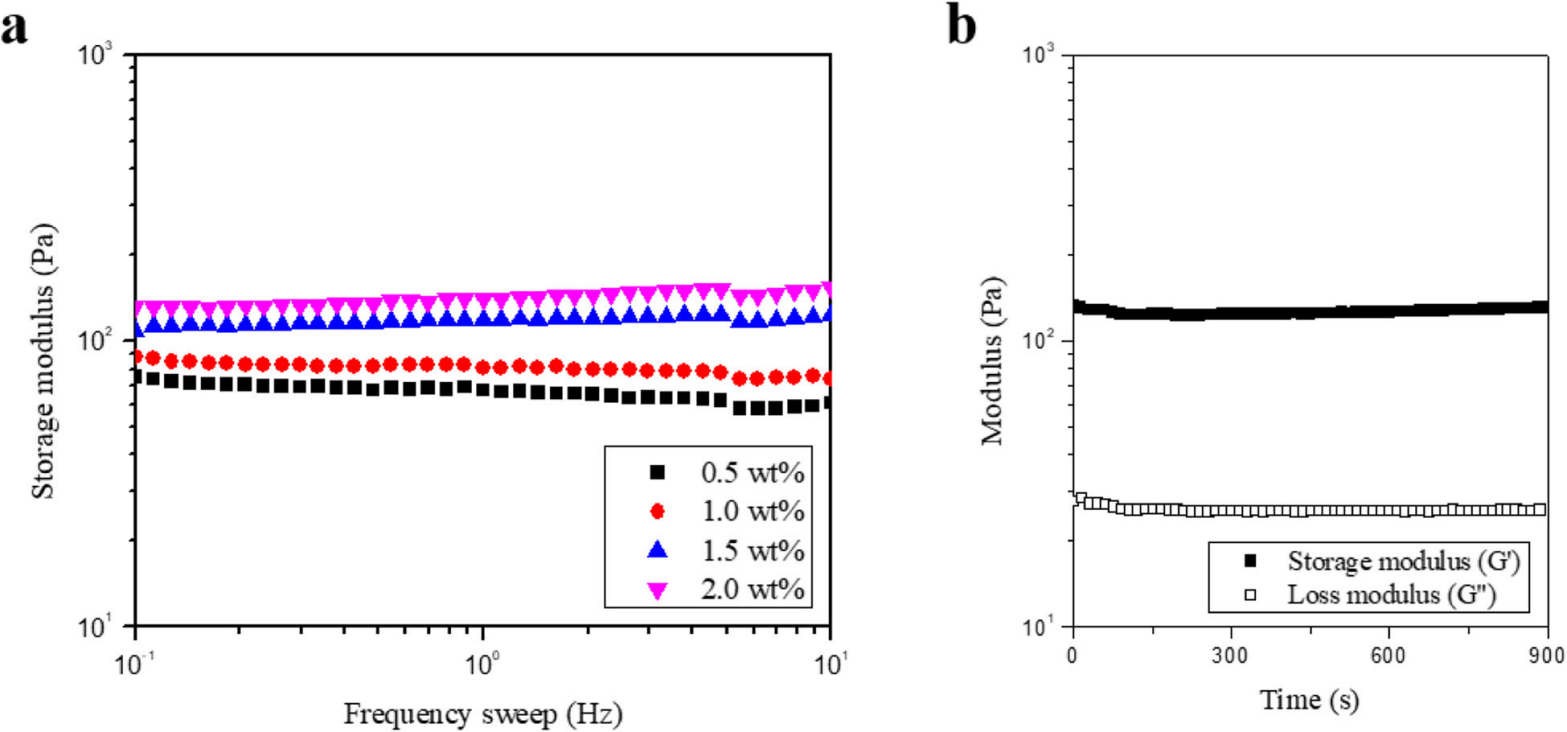
Rheological data of photocrosslinked HA/Tyr 25 hydrogels. **a** Frequency sweep of HA/Tyr 25 hydrogels formed with different polymer concentrations and (**b**) Time sweep of 2.0 wt% HA/Tyr 25 hydrogels under 0.5 mM RF, 100 mM SPS and 30 s irradiation condition

The rheological measurement was performed by comparing the storage modulus (G′) on various HA concentrations. The G′ is a reliable parameter to indicate how rigid the hydrogel is. As the HA concentration was increased from 0.5 wt% to 2.0 wt%, G′ value of HA/Tyr hydrogel was also increased (Fig. 5a). From the frequency sweep results, HA/Tyr 25 sample with 2.0 wt% concentration was chosen as optimal concentration for the hydrogelation under 0.5 mM RF, 100 mM SPS and 30 s irradiation conditions. In order to examine the gelation behavior of HA/Tyr 25 with 2.0 wt% concentration, the G′ and loss modulus (G″) were measured by a time sweep. In the time range from 0 to 900 s, the G′ remained higher than the G″, indicating that the 2.0 wt% HA/Tyr formed a stable hydrogel as a viscoelastic solid (Fig. 5b).

### Viscosity changes of HA solution in the presence of RF and SPS

**Fig. 6:**
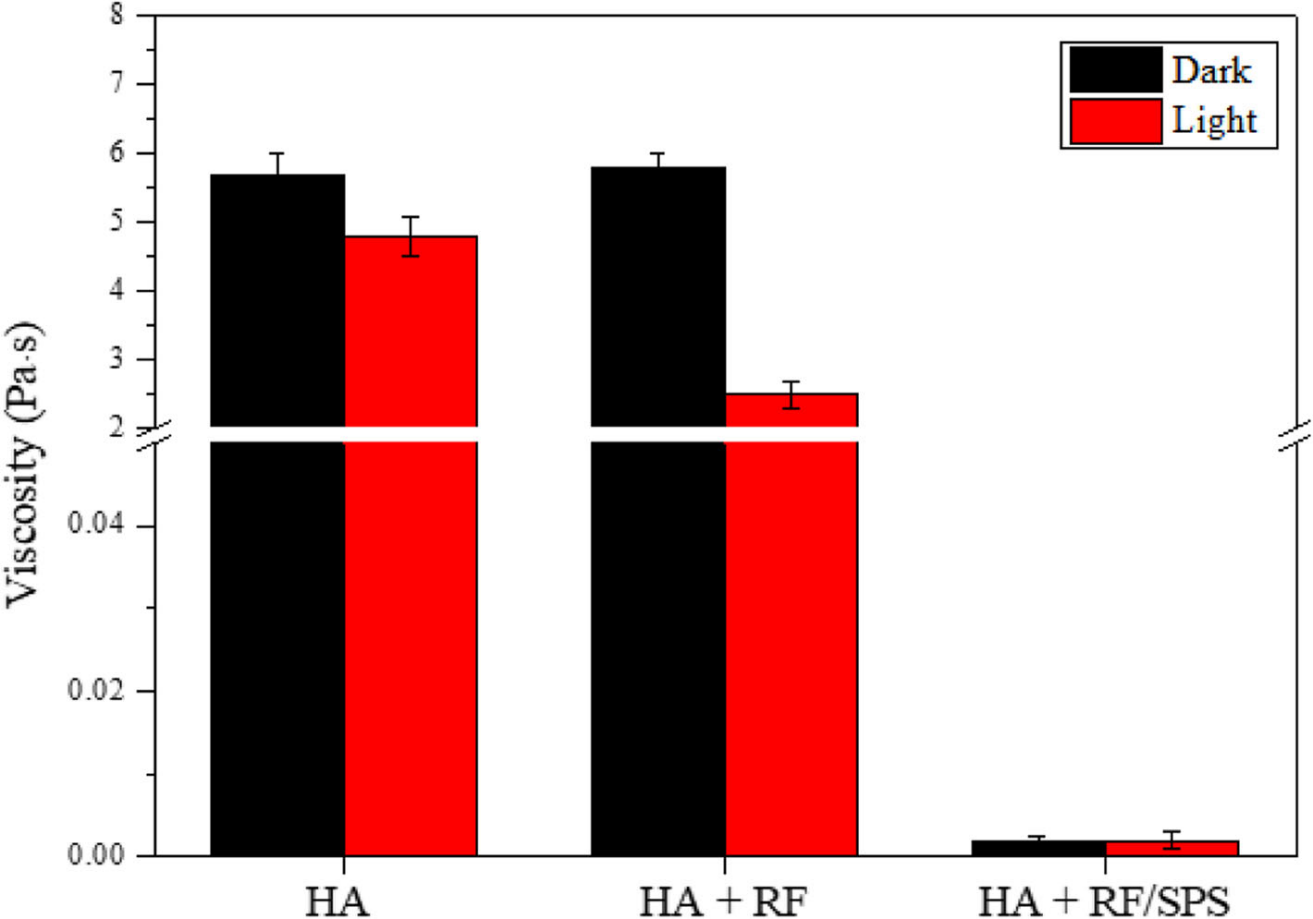
The effect of photo-excited RF and SPS on the viscosity of HA solution at light or dark condition

The effect of ROS generated by the photoinitiation reaction of RF was investigated by the viscosity change of HA solution using rheometer. The experiment was conducted with exposure of light in the presence of RF or RF/SPS. The effect of RF on HA molecule was shown in Fig. 6. Under the visible light condition, the viscosity of HA solution with RF was lower than that of HA solution without RF. Furthermore, the addition of SPS, which generate divalent anions, also considerably decreased the viscosity of HA solution. The decrease in the viscosity of HA solution was mainly associated with the degradation of HA molecular chains. The viscosity of HA solution with RF was decreased when visible light was irradiated. In contrast, the viscosity decrease of HA solution with RF was not observed under the dark condition. However, the addition of SPS induced a viscosity decrease, despite the dark condition (Fig. 6).

### Degradation of HA/Tyr hydrogels by ROS

**Fig. 7:**
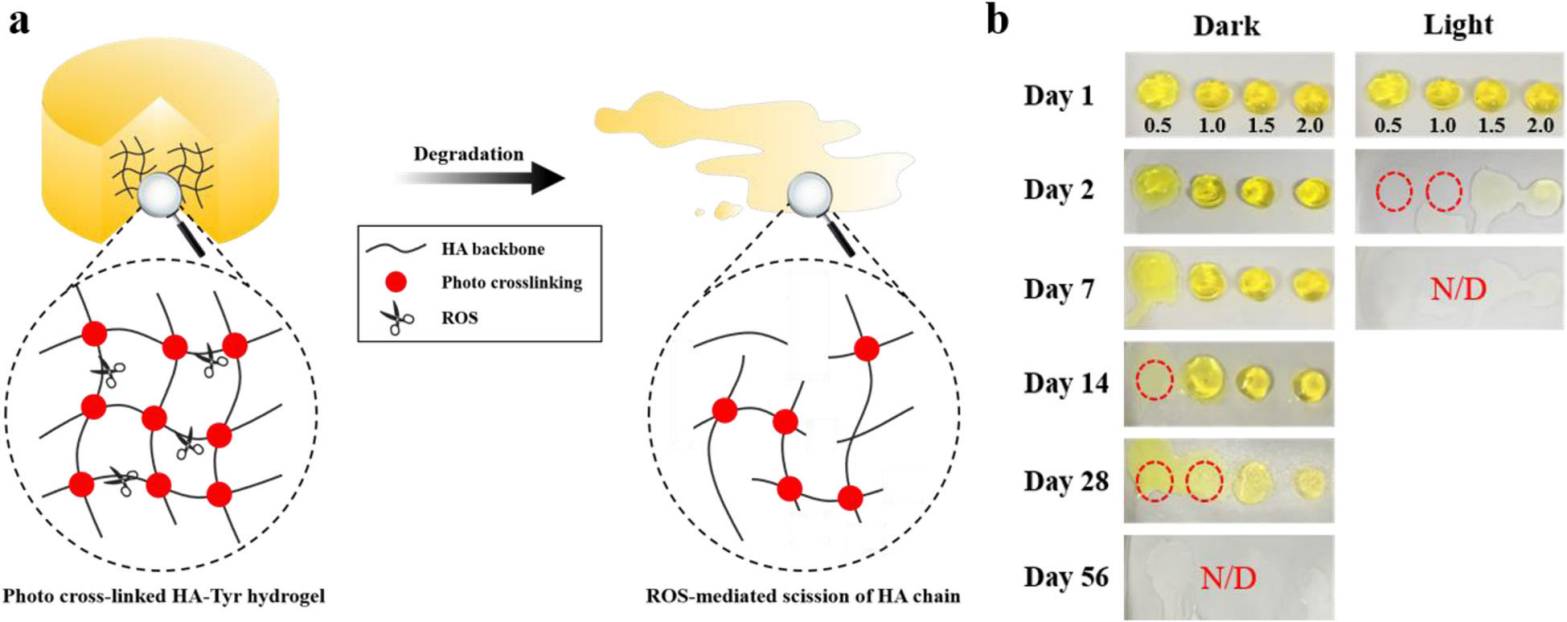
**a** Schematic presentaion on the degradation of HA/Tyr hydrogel by ROS derived from RF-based photochemistry. **b** Photographs of photocrosslinked HA/Tyr 25 hydrogel (0.5, 1.0, 1.5 and 2.0 wt%) showing the decomposition behavior over time under 0.5 mM RF, 100 mM SPS and 30 s irradiation time

Based on previous experiment, the degradation behavior of HA/Tyr 25 hydrogel was examined. The degradation rate of HA was faster under the visible light irradiation than under the dark condition, similar to the viscosity change result. In addition, the degradation rate was decreased with increasing HA concentration. Thus, HA/Tyr 25 hydrogel with 2.0 wt% concentration was stable in dark condition. This was attributed to the ROS generation during photoinitiation reaction of RF (Fig. 7a,b). In order to verify the HA degradation by ROS, HA/Tyr 25 hydrogel was washed with sodium azide, which is a ^1^O_2_ scavenger [26]. At 3 months after sodium azide treatment, the HA/Tyr hydrogel remained stable without a severe degradation.

## Discussion

Several studies on RF photochemistry were reported that RF (vitamin B_2_) can be reversibly reduced by hydrogen atoms by UV or visible light exposures [27–29]. In this study, RF-sensitized photocrosslinking system was applied to HA/Tyr hydrogel. RF as a photoinitiator induced the generation of oxygen radicals that mediate a covalent bond (crosslink) between phenolic moieties of tyramine. When exposed to light, RF absorbed energy, and turned into the triplet exited state (^3^RF*). It reacted with substrates (Type I) or oxygen molecular species (Type II), and generated ROS in this progress. The HA/Tyr crosslinking was induced by radical intermediates, such as superoxide anion radicals (O_2_^∙-^), which formed by ^3^RF*. The photocrosslinking of HA/Tyr conjugate was originated from this RF photochemistry. Rheological analysis showed that the storage modulus (G′) was increased with increasing polymer concentration, and this result was attributed to the increase in entanglement and reactive phenol group concentration.

The viscosity of HA solution was decreased in the presence of RF photoinitiator. The viscosity drop was mainly due to the degradation of HA molecules, as reported by Andley and Chakrabarti [30]. The degradation of HA was associated with the ROS generated by the photo-excited RF because the viscosity decrease was not observed in the dark condition or in the absence of RF.

In addition, when SPS was added, the viscosity of the HA solution was much lower than the addition of RF only. This result was due to divalent anions, such as sulfate and phosphate groups, which were known to catalyze the ROS generation in RF system [29]. Also, riboflavin phosphate, a water-soluble form of RF, was used as photoinitiator in this study. This might also influence the generation of ROS. However, when SPS was added, the viscosity decrease was observed even though in the dark condition. When SPS was dissolved in water, it formed persulfate free radicals, which is oxidant with similar oxidation potentials as hydroxyl radical (∙OH) [31, 32]. Thus, SPS was able to generate the degradation of HA molecule, regardless of the light.

Based on these results, HA/Tyr hydrogel was gradually degraded under visible light irradiation, and the degradation rate was decreased with increasing HA concentration. Degradation occurred slowly after washing with singlet oxygen scavenger solution, suggesting that HA degradation was closely related to ROS.

Consequently, RF-based, visible light-initiated photochemical reaction simultaneously induced the crosslinking and degradation reaction of HA/Tyr, and the degradation of HA/Tyr hydrogel could be minimized by controlling polymer and initiator concentration, the light source intensity, and sufficient washing process.

## Conclusion

In this study, the photocrosslinkable HA/Tyr conjugates were successfully prepared by EDC/NHS chemistry. The HA/Tyr solution with 2.0 wt% concentration could form elastic hydrogels through RF-induced photocrosslinking under visible light irradiation. Their gelation behaviors were investigated by tube inverting method and rheological analysis. HA/Tyr hydrogel with or without photoinitiator showed different degradation rates. The lower the polymer concentration was, the faster the degradation rate was. The RF-based, visible light-initiated photochemical reaction system was contributed to both crosslinking and degradation of HA/Tyr hydrogel.

## Data Availability

Data generated and analyzed in this study IS available from the corresponding author on request.

## Abbreviations

^1^H-NMR: Proton nuclear magnetic resonance
ATR-FTIR: Attenuated total reflectance infrared spectroscopy
BDDE: 1,4-butanediol diglycidyl ether
DS: Degree of substitution
DVS: Divinyl sulfone
ECM: Extracellular matrix
EDC: 1-Ethyl-3-(3-dimethylaminopropyl) carbodiimide hydrochloride
GAG: Glycosaminoglycan
HA: Hyaluronic acid
HA-Tyr: Tyramine-modified hyaluronic acid
NHS: N-Hydroxysulfosuccinimide
RF: Riboflavin
ROS: Reactive oxygen species
Ru (II): Ruthenium
SPS: Sodium persulfate
Tyr: Tyramine
UV: Ultraviolet
